# Evaluating the predictive value of clinical models for HBV-related hepatocellular carcinoma: A meta-analysis

**DOI:** 10.3389/fmed.2025.1529201

**Published:** 2025-02-21

**Authors:** Long Huang, Luhuai Feng, Yang Lu, Bobin Hu, Hongqian Liang, Aoli Ren, Hang Wang, Wenming He, Caifang Deng, Minghua Su, Jianning Jiang

**Affiliations:** ^1^Department of Infectious Diseases, The First Affiliated Hospital of Guangxi Medical University, Nanning, Guangxi, China; ^2^Key Laboratory of Early Prevention and Treatment for Regional High Frequency Tumor, (Guangxi Medical University), Ministry of Education, Nanning, Guangxi, China

**Keywords:** clinical prediction model, external validation, full management, chronic HBV infection, hepatic carcinoma

## Abstract

**Objectives:**

Chronic viral hepatitis B (CHB) is a prevalent liver disease with primary hepatic carcinoma (HCC) as a severe complication. Clinical prediction models have gained attention for predicting HBV-related HCC (HBV-HCC). This study aimed to evaluate the predictive value of existing models for HBV-HCC through meta-analysis.

**Design:**

Meta-analysis.

**Data sources:**

Embase, PubMed, the Chinese Biomedical Literature Service System, and the Cochrane database were used for searches between 1970 and 2022.

**Methods:**

A meta-analysis was conducted to assess original studies on HBV-HCC prediction models. The REACH-B, GAGHCC, and CUHCC models were externally validated in a Guangxi cohort. The C-index and calibration curve evaluated 5 years predictive performance, with subgroup analysis by region and risk bias.

**Results:**

After screening, 27 research articles were included, covering the GAGHCC, REACH-B, PAGE-B, CU-HCC, CAMD, and mPAGE-B models. The meta-analysis indicated that these models had moderate discrimination in predicting HCC risk in HBV-infected patients, with C-index values from 0.75 to 0.82. The mPAGE-B (0.79, 95% CI: 0.79–0.80), GAG-HCC (0.80, 95% CI: 0.78–0.82), and CAMD (0.80, 95% CI: 0.78–0.81) models demonstrated better discrimination than others (*P* < 0.05), but most studies did not report model calibration. Subgroup analysis suggested that ethnicity and research bias might contribute to differences in model discrimination. Sensitivity analysis indicated stable meta-analysis results. The REACH-B, GAGHCC, CUHCC, PAGE-B, and mPAGE-B models had average predictive performance in Guangxi, with medium to low 3 and 5 years HCC risk prediction discrimination.

**Conclusion:**

Existing models have predictive value for HBV-infected patients but show geographical limitations and reduced effectiveness in Guangxi.

## 1 Introduction

Chronic hepatitis B virus (HBV) infection is one of the major public health problems worldwide and an important cause of hepatocellular carcinoma (HCC) (1), accounting for approximately all causes of HCC. More than 50% (2). Primary hepatocellular carcinoma ranks fourth in cancer-related mortality worldwide and is a disease that seriously threatens people’s lives and health (1). Chronic hepatitis B virus is closely related to the occurrence and development of HCC. Hepatitis-cirrhosis-liver cancer are typical disease development processes. Studies have shown that patients with chronic hepatitis B (CHB) have a lifetime risk of liver cancer of 25–40% (3), and the risk of HCC in patients with cirrhosis is doubled compared with patients without cirrhosis (4, 5). Effective antivirals can significantly reduce the incidence of HCC, but only approximately one-third of chronic hepatitis B patients with cirrhosis and half of patients with hepatocellular carcinoma receive antiviral treatment (3). Although nucleoside analogs have been shown to inhibit hepatitis B virus replication and reduce the risk of HCC, they cannot completely prevent the development of HCC. If HCC can be detected and diagnosed early during the treatment and monitoring of CHB patients, patients will have more treatment options, the probability of clinical cure will be greatly improved, and the long-term prognosis of patients will definitely be improved. Therefore, it is crucial to identify and closely monitor patients at high risk for HCC.

Although the existing guidelines for CHB treatment recommend that CHB patients undergo tests for liver biochemical indicators, HBV DNA quantification, HBV serum virological markers, liver stiffness, AFP, abdominal B-ultrasound, liver CT, and MRI every 3–6 months during antiviral treatment (6–8). However, due to the poor compliance of most patients, low sensitivity of examinations, high costs, and lax treatment standards in various regions, the early diagnosis and treatment of HCC in CHB patients in my country still faces huge challenges (9). Therefore, screening out high-risk groups for liver cancer in the CHB population and regular monitoring and follow-up is an economical and effective method to achieve early diagnosis and treatment of HCC. The disease risk prediction model is a risk assessment tool for achieving individualized prediction. It can dynamically estimate the probability of HCC in CHB patients, thereby helping doctors improve the compliance of CHB patients during treatment and the implementation of HCC screening and monitoring strategies (10). Currently, several prediction models have been used to predict the risk of HCC in CHB patients (11–16), including REACH-B, GAG-HCC, CUHCC, mPAGE-B, PAGE-B and CAMD. However, all of them need to be based on specific disease or status backgrounds, such as patients in the cirrhosis stage, strict entecavir or tenofovir antiviral treatment years, etc., which is not conducive to further expansion of application in the real world and it is difficult to carry out unified monitoring and stratified management of chronic liver disease patients across regions. Therefore, none of them are recommended by the guidelines for widespread use in clinical practice.

During the development and validation of prediction models, limited sample size and outcome events may lead to conflicting evidence and relatively low statistical power (17). Therefore, it is necessary to synthesize the evidence from all external validation studies of the same model to evaluate the performance of the model in different populations with relatively large sample sizes. In addition, to date, there is little evidence comparing the performance of all different HCC prediction models in a head-to-head validation in the same cohort. In this study, we aimed to systematically identify all published prediction models for the risk of HCC in patients with CHB and perform external validation of all models by meta-analysis. We then used our long-term follow-up cohort of CHB patients to perform independent external validation of existing commonly used models (including REACH-B, GAG-HCC, CUHCC, mPAGE-B, PAGE-B, and CAMD models) to evaluate their clinical applicability.

## 2 Methods

This study was conducted in accordance with the 2020 Standard Reporting Items for Systematic Reviews and Meta-Analyses (18).

### 2.1 Inclusion and exclusion criteria

#### 2.1.1 Inclusion criteria

(1) The subjects were patients with chronic HBV infection (including chronic hepatitis B virus hepatitis and hepatitis B cirrhosis);
(2) The prediction model was constructed and validated by multivariate analysis of commonly used clinical parameters, such as clinical diagnosis, age, gender, family history of HCC, history of antiviral treatment (including antiviral drugs and time), HBeAg status, HBV DNA, platelets (PLT), alanine aminotransferase (ALT), total bilirubin (TBil), albumin (ALB), aspartate aminotransferase (AST), and AFP;
(3) The study reported the predictive performance of the model for HCC development, including the discrimination and/or calibration of the model;
(4) No language restrictions;
(5) The risk prediction model has been externally validated (applied) > 5 times (17).

#### 2.1.2 Exclusion criteria

(1) Abstracts, case reports, reviews, meta-analyses, systematic reviews, and non-clinical research literature;
(2) Use of literature with duplicate research data or duplicate reports.

### 2.2 Literature search strategy

Original studies on clinical prediction models for predicting HBV-HCC risk were retrieved through computer retrieval of Embase, PubMed, China Biomedical Literature Service System, and Cochrane database (time: 1970–2022). In addition, we also checked the reference lists of all included articles to supplement the acquisition of other relevant research literature. According to the strategy of combining subject terms and free word searches, the details of search strategy were provided in Supplementary material.

### 2.3 Data extraction and literature quality evaluation criteria

Data were extracted from the studies according to the critical assessment and data extraction checklist for systematic reviews of predictive model studies (19). The extracted information included: authors, year of publication, country, sample size of the study, and number of HCC cases included in the study; age, gender, follow-up time, and laboratory test data of the study population (including whether antiviral treatment was used, proportion of HBeAg-positive patients, proportion of patients with cirrhosis, HBV DNA, ALB, ALT, PLT, TBil, and AFP); and the model’s discrimination (C-index and 95% confidence interval) and calibration, i.e., the ratio of the number of observed [observed (O)] HCC cases to the number of expected [expected (E)] HCC cases (O:E).

The prediction model risk of bias assessment tool (PROBAST) was used to assess the bias risk of the included literature. The PROBAST tool contains 20 items in four areas: research subjects, predictive factors, outcomes, and statistical analysis (20, 21). The quality of the literature is divided into three levels: unclear, low risk of bias, and high risk of bias.

### 2.4 External validation cohort

A total of 261 patients with chronic HBV infection who visited the First Affiliated Hospital of Guangxi Medical University from 2005 to 2023 were selected to verify the 3 and 5 years HCC prediction performance of the HCC risk model included in the meta-analysis. The clinical characteristics of all chronic HBV-infected patients were collected during the cohort follow-up to verify the HCC risk model, including demographic characteristics: age, gender, family history of HCC; laboratory test results: including serum ALT, TBil, albumin (ALB), HBeAg, HBV DNA, platelets (PLT). At the beginning of follow-up, liver puncture biopsy was performed on patients who needed it to assess the degree of liver fibrosis, and abdominal ultrasound or CT and liver angiography were monitored for 3–6 months during the follow-up to assess the changes in intrahepatic lesions. At the same time, the HBV-HCC risk model included in the meta-analysis was cross-sectionally validated.

Diagnostic criteria for CHB (6): (1) serum HBsAg positive; (2) B-ultrasound suggests chronic hepatitis without nodules; (3) Fibroscan liver stiffness < 12.4 kPa. Diagnostic criteria for LC (6): The following conditions must be met for the diagnosis of hepatitis B-related cirrhosis: (1) and (2) or (1) and (3). Among them, (1) the patient is currently HBsAg positive, or HBsAg negative, anti-HBc positive and has been HBsAg positive for more than 6 months, and other causes are excluded; (2) liver biopsy pathology shows characteristics of cirrhosis; (3) clinical diagnosis, at least two of the following five items are met, and non-cirrhotic portal hypertension is excluded: ② imaging examination reveals signs of cirrhosis and/or portal hypertension; ③ endoscopic examination reveals esophageal and gastric varices; ④ liver stiffness measurement (LSM) shows cirrhosis (when ALT < 1 × ULN, LSM ≥ 12.0 kPa; when 1 × ULN < ALT < 5 × ULN, LSM ≥ 17.0 kPa); ⑤ serum ALB level is less than 35 g/L and/or prothrombin time (PT) is prolonged by more than 3 s compared with the control; ⑥ platelet count is less than 100 × 10^9/L.

Exclusion criteria: (1) patients with other infectious diseases such as hepatitis C, hepatitis D, AIDS, and other malignant tumors; (2) patients with other liver diseases such as drug-induced liver disease, alcoholic liver disease, and autoimmune liver disease.

### 2.5 Statistical analysis

All statistical analyzes were completed using Review Manager 5.4.1 and Stata 17.0 software. External validation studies of HCC risk prediction models that met the inclusion criteria were included in the meta-analysis. Use the random effects model to analyze the AUC or C-index of the summary model to measure the discrimination of the model, that is, the accuracy of the model in distinguishing the presence or absence of a disease (such as liver cancer). The range of AUC or C-index is 0∼1, AUC or C -The closer the index value is to 1, the higher the accuracy of the model. The O:E value measures the calibration of the model (22, 23) and evaluates the consistency between the results predicted by the model and the actual results. Ideally, the O:E value of a model is close to 1, which means that the results predicted by the model are very consistent with the actual situation. consistent. The Z test compares whether there is a significant difference in the total discrimination of each model; I^2^ quantifies the degree of difference between the results of different studies in meta-analysis. Its value ranges from 0 to 100%. An I^2^ value greater than 50% is usually considered a study. There is high heterogeneity among the studies, which means that the differences between different studies are large, and the simple combined results may not be accurate enough or need to be interpreted with caution. Subgroup and sensitivity analyses were performed stratified by region (Asian or non-Asian) and risk of bias (high risk, unclear, or low risk) to identify possible sources of heterogeneity. Funnel plot, Begg test and Egger test were used to determine whether there was publication bias.

In the validation cohort, categorical variables were expressed as *n* (%), and continuous variables were expressed as mean ± standard deviation or median (interquartile range) depending on whether they conformed to normal distribution. Because we lacked data on the history of diabetes and some patients lacked follow-up records of platelet count results, we only performed time-dependent (longitudinal) external validation in REACH-B, GAGHCC, and CUHCC. The risk of HCC was calculated according to the HCC risk prediction model calculation formula, and the time-dependent and time-independent (cross-sectional) discrimination and 95% confidence intervals included in this meta-analysis model were calculated, and calibration curves were drawn to evaluate the calibration of the model. The prediction time points of all models were limited to 3 and 5 years.

## 3 Results

### 3.1 Search results and literature characteristics

**FIGURE 1:**
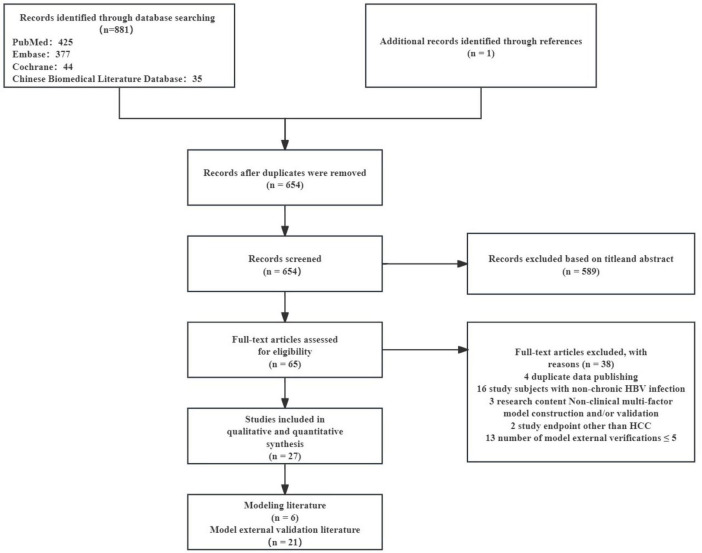
Flowchart of literature search. HBV, hepatitis B virus; HCC, hepatocellular carcinoma.

As shown in Figure 1, a total of 881 articles were retrieved from the four databases. Combined with the exclusion criteria, 27 articles were included in the final meta-analysis. The characteristics are shown in Table 1, including 21 articles on modeling and external validation of six HCC risk prediction models. The prediction models that have been externally validated more than five times are PAGE-B model (15), mPAGE-B model (14), CUHCC model (13), REACH-B model (11), GAG-HCC model (12), and CAMD model (16). The PAGE-B model has been externally validated 21 times (a total of 63,041 HBV-infected people were included, including 3,540 HCC patients), the mPAGE-B model has been externally validated 15 times (a total of 55,664 HBV-infected people were included, including 3,097 HCC patients), the CUHCC model has been externally validated 11 times (a total of 13,944 HBV-infected people were included, including 651 HCC patients), the REACH-B model has been externally validated 10 times (a total of 14,911 HBV-infected people were included, including 710 HCC patients), the GAG-HCC model has been externally validated seven times (a total of 10,709 HBV-infected people were included, including 571 HCC patients), and the CAMD model has been externally validated seven times (a total of 32,922 HBV-infected people were included, including 1,046 HCC patients). The modeling and validation research literature of the HCC risk model for HBV-infected people is shown in Table 2.

**TABLE 1:** Characteristics of studies included in meta-analysis.

	The first author	Model	Type	Year	Nation	Sample size	HCC (*n*, %)	HCC diagnostic methods
1	Yang HI	REACH-B	Modeling	2011	China	1,505	111 (7.4%)	Pathological or clinical diagnosis
2	Kim JH	mPAGE-B, PAGE-B, REACH-B, CUHCC, GAG-HCC	Modeling + Verify	2018	Korea	1,000	72 (7.2%)	Clinical diagnosis
3	Kim HY	REACH-B, CUHCC, mPAGE-B, PAGE-B	Verify	2022	Korea	1,640	112 (6.8%)	Clinical diagnosis
4	Lee HW	mREACH-B	Verify	2019	Korea	1,330	128 (9.6%)	Unknown
5	Kim MN	PAGE-B, REACH-B, CUHCC, GAG-HCC	Verify	2017	Korea	1,092	36 (3.3%)	Pathological or clinical diagnosis
6	Abu-Amara M	REACH-B, CUHCC, GAG-HCC	Verify	2015	Canada	2,105	70 (3.3%)	Pathological or clinical diagnosis
7	Kim HS	REACH-B, CUHCC, mPAGE-B, PAGE-B, CAMD	Verify	2021	United States	3,101	113 (3.6%)	Unknown
8	Kamalapirat T	mPAGE-B, PAGE-B, REACH-B, CUHCC, GAG-HCC, CAMD	Verify	2010	Thailand	2,208	20 (0.9%)	Clinical diagnosis
9	Costa APM	REACH-B, PAGE-B	Verify	2022	Brazil	978	34 (3.5%)	Pathological or clinical diagnosis
10	Brouwer WP	REACH-B, GAG-HCC, CUHCC, PAGE-B	Verify	2017	Nether-lands	557	40 (7.2%)	Pathological or clinical diagnosis
11	Yuen MF	GAG-HCC	Modeling + Verify	2009	China	820	52 (6.3%)	Pathological or clinical diagnosis
12	Ji JH	GAG-HCC, mPAGE-B, PAGE-B, CAMD	Verify	2021	Korea	1,763	163	Pathological or clinical diagnosis
3	Wong VW	CUHCC	Modeling + Verify	2010	China	424	45 (10.6%)	pathological or Clinical diagnosis
14	Wong GL	CUHCC	Verify	2013	China	520	17 (3.4%)	Pathological or clinical diagnosis
15	Zhang JC	mPAGE-B, PAGE-B	Verify	2022	China	707	57 (8.1%)	Pathological or clinical diagnosis
16	Yip TCF	mPAGE-B, PAGE-B	Verify	2019	China	32,150	1,532 (4.8%)	Unknown
17	Papatheod-oridis GV	PAGE-B	Verify	2021	Greece	1,951	142 (7.2%)	Pathological or clinical diagnosis
18	Lee JS	PAGE-B	Verify	2021	Korea	2,037	182 (8.9%)	Pathological or clinical diagnosis
19	Kim SU	mPAGE-B, PAGE-B, CAMD	Verify	2019	Korea	3,277	292 (8.9%)	Pathological or clinical diagnosis
20	Güzelbulut F	mPAGE-B, PAGE-B, CAMD	Verify	2021	Turkey	647	26 (4.0%)	Unknown
21	Ferreira da Silva AC	mPAGE-B, PAGE-B	Verify	2022	Brazilian	224	15 (6.7%)	Pathological or clinical diagnosis
22	Chon HY	mPAGE-B, PAGE-B	Verify	2021	Korea	973	42 (4.3%)	Pathological or clinical diagnosis
23	Chang JW	mPAGE-B, PAGE-B	Verify	2020	Korea	3,171	280 (8.8%)	Pathological or clinical diagnosis
24	Papatheod-oridis G	PAGE-B	Modeling + Verify	2016	Canada	490	41 (8.4%)	Pathological or clinical diagnosis
25	Seo YS	PAGE-B	Verify	2017	Korea	1,241	66 (5.3%)	Pathological or clinical diagnosis
26	Nguyen MH	PAGE-B	Verify	2017	China	2,683	203 (7.5%)	Pathological or clinical diagnosis
27	Hsu YC	CAMD	Modeling + Verify	2018	China	19,321	383 (1.98%)	Unknown

**TABLE 2:** Research literature on the development and validation of risk models for hepatocellular carcinoma (HCC) in hepatitis B virus (HBV)-infected patients.

Predictive model	Model modeling research	Model validation study
REACH-B	Yang HI(11)	Kim, J. H(14), Kim HY(28), Yang, H. I(11), Lee, H. W(26), Kim, M. N(29), Abu-Amara M(30), Kim, H. S(31), Kamalapirat, T(32), Costa, A. P. M(33), Brouwer, W. P(34)
GAG-HCC	Yuen MF(12)	Kim, J. H(14), Lee, H. W(26), Kim, M. N(29), Abu-Amara M(30), Kamalapirat, T(32), Ji, J.H(35), Brouwer, W. P(34)
CUHCC	Wong VW(13)	Wong VW(13), Kim, J. H(14), Kim HY(28), Wong, G.L(36), Lee, H. W(26), Kim, M. N(29), Abu-Amara M(30), Kim, H. S(31), Kamalapirat, T(32), Brouwer, W. P(34)
mPAGE-B	Kim, J. H(14)	Zhang JC (37), Yip, T. C. F(38), Papatheodoridis, G. V(39), Lee, J. S(40), Lee, H. W(26), Kim, S. U(41), Kim, J. H(14), Kim, H. S(31), Kamalapirat, T(32), Ji, J. H(35), Güzelbulut, F(42), Ferreira da Silva, A.C(43), Chon H. Y(44), Chang, J. W(45), Kim HY(28)
PAGE-B	Papatheodoridis G(15)	Junchao Zhang (37), Kim, J. H(14), Yip, T. C. F(38), Seo, Y. S(46), Papatheodoridis, G. V(39), Nguyen, M.H(47), Lee, J. S(40), Lee, H. W(26), Kim, S. U(41), Kim, M. N(29), Kim, H. S(31), Kamalapirat, T(32), Ji, J. H(35), Güzelbulut, F(42), Ferreira da Silva, A. C.(43), Costa, A. P. M(33), Chon HY(44), Chang, J. W(45), Brouwer, W. P(34), Papatheodoridis, G(15), Kim H. Y(28)
CAMD	Hsu YC(16)	Papatheodoridis, G. V(39), Kim, S. U(41), Kim, H. S(31), Kamalapirat, T(32), Ji, J. H(35), Hsu Y. C (16), Güzelbulut, F(42)

The modeling population of the HCC risk prediction model is mainly Asian (China and South Korea) and North American (Canada) races, and the validation population covers multiple regions in South America, Europe, and Asia, with Asia as the main region. The number of predictive factors in the model is 3–5, which is mainly used to predict the risk of HCC in HBV-infected patients at 3, 5, and 10 years (as shown in Table 3). Different models are used to predict HBV-infected patients with specific disease states. The mPAGE-B, PAGE-B, and CAMD models are suitable for chronic hepatitis B patients treated with entecavir or tenofovir antiviral therapy, the CUHCC model is suitable for chronic hepatitis B patients in all disease states, the GAG-HCC model is only suitable for chronic hepatitis B patients not treated with antiviral therapy, and REACH-B is suitable for chronic hepatitis B patients treated with antiviral therapy and without cirrhosis. Age is the most common predictive factor in all models, followed by gender (five models), cirrhosis (three models), and HBV DNA (three models).

**TABLE 3:** Predictor variables included in the six hepatocellular carcinoma (HCC) risk prediction models for hepatitis B virus (HBV)-infected patients.

Predictor	HCC risk prediction model					
	**REACH-B**	**GAG-HCC**	**CUHCC**	**mPAGE-B**	**PAGE-B**	**CAMD**
Age	●	●	●	●	●	●
Gender	●	●	–	●	●	●
Cirrhosis	–	●	●	–	–	●
Diabetes	–	–	–	–	–	●
HBeAg	●	–	–	–	–	–
HBV DNA	●	●	●	–	–	–
ALT	●	–	–	–	–	–
PLT	–	–	–	●	●	–
ALB	–	–	●	●	–	–
TBIL	–	–	●	–	–	–

●Indicates that the predictor is included in the HCC risk prediction model.

### 3.2 Literature quality assessment

**FIGURE 2:**
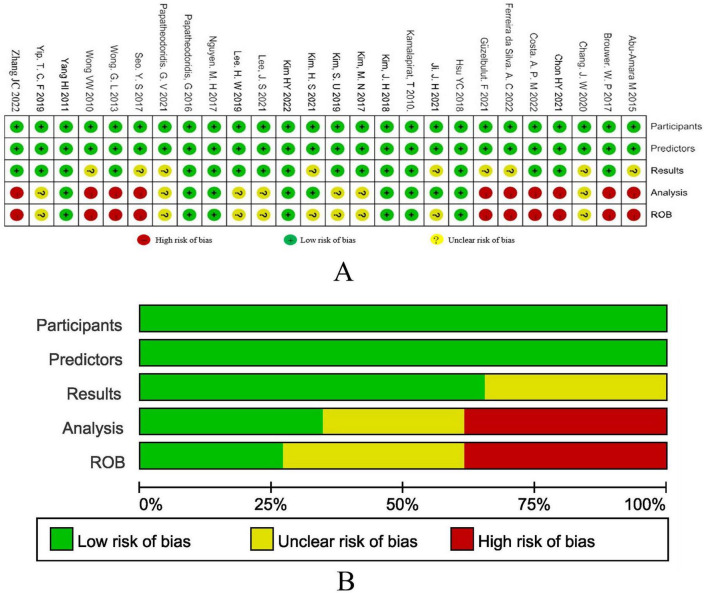
**(A)** The results of bias risk assessment of the research subjects, predictors, results and analysis methods of the included studies according to the PROBAST criteria. **(B)** Risk of bias assessment results of included studies summarized according to PROBAST guidelines.

Figure 2 show the results of the research quality assessment of the 27 articles included in the meta-analysis based on the PROBAST tool. A total of 10 (37.0%) studies had a high risk of bias, 9 (33.3%) studies had incomplete information, and 8 (29.6%) studies had a low risk of bias. The high risk of bias in the studies was mainly due to the fact that the sample size of the analysis part was less than 100 outcome events, there was no description of the treatment of missing data, and no calculation of model calibration.

### 3.3 Meta-analysis results

In the meta-analysis, all external validation studies of the model except the CAMD model reported the discrimination (C-index value) of the 5 years HCC risk in HBV-infected patients, but the number of assessments of the 3 and 10 years HCC risk was less than five. Only three external studies reported the 3 years HCC risk in the REACH-B model, and one external study reported the 10 years HCC risk; only one external study reported the 10 years HCC risk in the CUHCC and GAG-HCC models; the external validation studies of the CAMD model reported the discrimination (C-index value) of the 3 years HCC risk in HBV-infected patients, and only one study reported the 1 and 2 years HCC risks. Since only a small number of articles (two articles) reported the O:E value, a summary analysis of the model calibration could not be performed in this study. To improve the accuracy of the Meta-analysis results, this study only analysed the C-index values of the REACH-B, GAG-HCC, CUHCC, mPAGE-B, and PAGE-B models for predicting the 5 years HCC risk and the C-index value of the CAMD model for predicting the 3 years HCC risk in HBV-infected patients. The results are shown in Table 4. The discrimination of all models was at a moderate level, with C-index values ranging from 0.75 to 0.82. Among them, the discrimination of the mPAGE-B, GAG-HCC, and CAMD models was better than that of other models (*P* < 0.05), but there was no significant statistical difference in the discrimination among the three models (*P* > 0.05). The Meta-analysis results of all models showed significant heterogeneity.

**TABLE 4:** Discrimination of six models in predicting hepatitis B virus (HBV)-related hepatocellular carcinoma (HCC) risk.

Model	Number of applications	C-index	C-index 95% CI
REACH-B	10	0.71	0.70–0.73
GAG-HCC	7	0.80	0.78–0.82
CUHCC	11	0.76	0.75–0.78
mPAGE-B	15	0.79	0.79–0.80
PAGE-B	21	0.77	0.76–0.77
CAMD	7	0.80	0.78–0.81

### 3.4 Subgroup analysis

The results of all subgroup analysis are shown in Tables 5, 6. In the regional subgroup analysis (Table 5), except for the REACH-B model, the GAG-HCC, CUHCC, mPAGE-B, PAGE-B and CAMD models were all less capable of distinguishing the risk of HCC in Asian populations than in non-Asian populations. crowd (*P* < 0.05). The discrimination of the mPAGE-B model in predicting the 5 years risk of HCC in the Asian population (C-index: 0.79; 95% confidence interval: 0.78–0.80) is better than that of the REACH-B, GAG-HCC, CUHCC, and PAGE-B models. High (*P* < 0.05), but there is significant heterogeneity in the studies of Asian populations included in the REACH-B, CUHCC, mPAGE-B and PAGE-B models, while the GAG-HCC model has significant heterogeneity in non-Asian populations. There was significant heterogeneity across studies. The discrimination degrees of each model in predicting the 5 years risk of HCC in non-Asian populations were similar (*P* > 0.05).

**TABLE 5:** Summary of discrimination of different models in regional subgroup analysis.

Model	Subgroup	Number of applications	C-index	95% CI	I^2^	*P*
REACH-B	Asia	5	0.71	0.70–0.73	83.20%	0.97
	No-Asia	5	0.71	0.70–0.73	16.80%	
GAG-HCC	Asia	4	0.76	0.73–0.79	46.80%	< 0.001
	No-Asia	3	0.83	0.81–0.86	53.20%	
CUHCC	Asia	6	0.74	0.72–0.76	72.50%	< 0.001
	No-Asia	5	0.82	0.80–0.85	27.50%	
mPAGE-B	Asia	9	0.79	0.78–0.80	90.80%	0.001
	No-Asia	6	0.82	0.80–0.84	9.20%	
PAGE-B	Asia	11	0.76	0.75–0.77	81.60%	< 0.001
	No-Asia	10	0.8	0.79–0.82	18.40%	
CAMD	Asia	3	0.77	0.75–0.79	49.30%	< 0.001
	No-Asia	4	0.82	0.80–0.81	50.70%	

**TABLE 6:** Summary of discrimination of different models in subgroup analysis of risk of bias.

Model	Subgroup	Number of applications	C-index	95% CI	I^2^	*P*
REACH-B	High	3	0.77	0.73–0.81	11.50%	–
	Unclear	3	0.64	0.60–0.67	14.80%	< 0.001
	Low	4	0.72	0.70–0.73	73.70%	–
GAG-HCC	High	2	0.85	0.81–0.89	21.10%	–
	Unclear	3	0.79	0.77–0.82	66.80%	0.001
	Low	2	0.73	0.67–0.78	12.10%	–
CUHCC	High	4	0.82	0.79–0.86	18.50%	–
	Unclear	4	0.73	0.70–0.76	25.30%	< 0.001
	Low	3	0.75	0.74–0.77	56.30%	–
mPAGE-B	High	4	0.82	0.80–0.85	7.20%	–
	Unclear	8	0.79	0.78–0.79	57.70%	0.001
	Low	3	0.80	0.79–0.81	35.10%	–
PAGE-B	High	7	0.82	0.80–0.84	10.80%	–
	Unclear	9	0.76	0.75–0.77	71.20%	< 0.001
	Low	5	0.76	0.75–0.78	18.00%	–
CAMD	High	1	0.87	0.84–0.90	21.20%	< 0.001
	Unclear	4	0.79	0.77–0.80	57.90%	–
	Low	3	0.75	0.72–0.78	20.90%	–

The results of bias risk subgroup analysis showed (Table 6) that the REACH-B, GAG-HCC, CUHCC, mPAGE-B, PAGE-B, and CAMD models had higher discrimination of HCC risk in high-risk bias studies than those in unclear bias risk group and low-risk bias group (*P* < 0.05). In the high-risk bias subgroup, the discrimination of GAG-HCC model in predicting 5 years HCC risk (C-index: 0.85; 95% confidence interval: 0.81–0.89) was higher than that of GAG-HCC, CUHCC, mPAGE-B and PAGE-B models (*P* < 0.05). In the subgroup with unclear risk of bias, the discrimination of REACH-B model in predicting the 5 years risk of HCC (C-index: 0.64; 95% confidence interval: 0.60–0.67) was lower than that of GAG-HCC, CUHCC, mPAGE-B, and PAGE-B models (*P* < 0.05). In the subgroup with low risk of bias, the discrimination of mPAGE-B model in predicting the 5 years risk of HCC (C-index: 0.85; 95% confidence interval: 0.81–0.89) was higher than that of GAG-HCC, CUHCC, GAG-HCC, and PAGE-B models (*P* < 0.05).

### 3.5 Sensitivity analysis and publication bias

**FIGURE 3:**
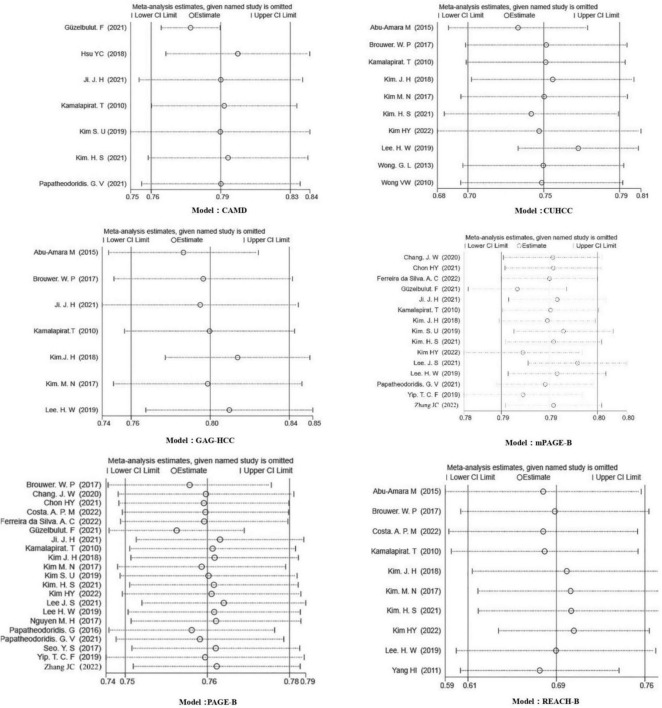
Model sensitivity analysis chart.

**FIGURE 4:**
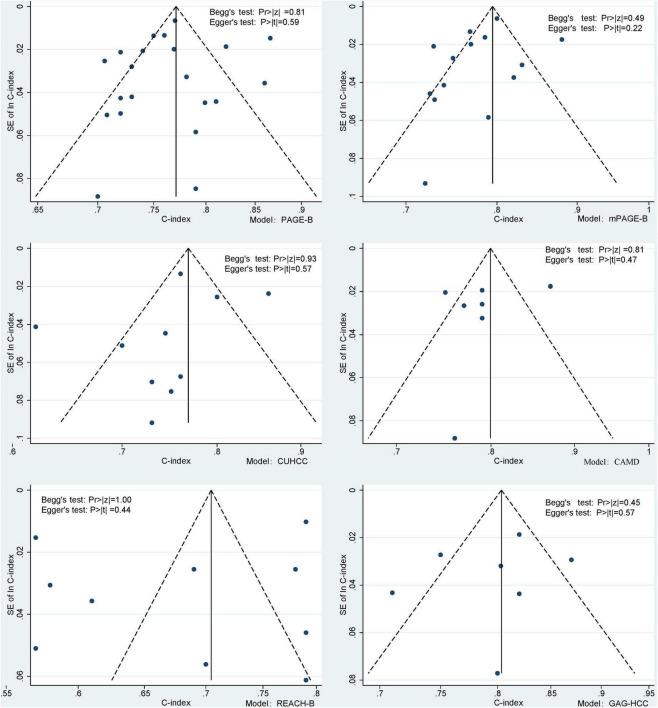
Model bias risk diagram.

The sensitivity analysis in each prediction model showed (Figure 3) that after eliminating one study at a time, the 95% confidence interval of the random effect model of the overall effect value of each model fluctuated within the 95% confidence interval of the original overall effect value, indicating that our meta-analysis results were stable. In the publication bias analysis, the funnel plots of each model are shown in Figure 4. The Begg test Pr > | z| values were all greater than 0.05, and the Egger test Pr > | t| values were all greater than 0.05, indicating that there was no publication bias.

### 3.6 Clinical characteristics and prediction model performance of the chronic HBV infection cohort in Guangxi

In the validation cohort of chronic HBV infection patients in Guangxi, 201 (77%) males and 60 (23%) females of 261 patients received nucleotide analog antiviral treatment, including 138 (52.9%) CHB patients, 76 (29.1%) LC patients and 47 (18.0%) patients pathologically diagnosed with HCC after partial liver resection. The median follow-up time was 120 months. The 3 years cumulative incidence of HCC was 4%, and the 5 years cumulative incidence was 11%.

**FIGURE 5:**
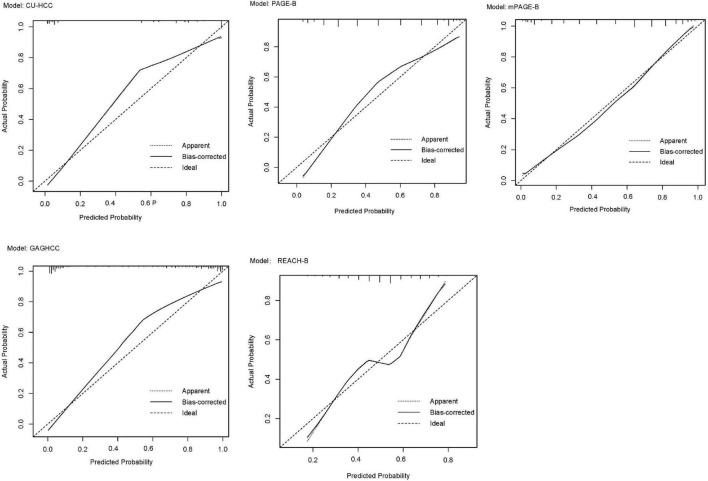
Calibration curve of REACH-B **(A)**, GAGHCC **(B)** and CUHCC **(C)** models predicting 5 years hepatocellular carcinoma (HCC) risk.

External validation was performed in REACH-B, GAGHCC, and CUHCC, and the time-dependent discrimination was calculated. The results are shown in Table 7. The ability of the three models of REACH-B, GAGHCC, and CUHCC to predict the risk of HBV-HCC in patients with chronic HBV infection in Guangxi was significantly lower than the meta-analysis summary results, and the 3 years and 5 years HCC risk prediction discrimination was at a medium-low level. The calibration curves showed (Figure 5) that the three models of REACH-B, GAGHCC, and CUHCC overestimated the risk of liver cancer in patients with chronic HBV infection in Guangxi.

**TABLE 7:** Discrimination of REACH-B, GAG-HCC, and CUHCC prediction models in the Guangxi chronic hepatitis B virus (HBV) infection patients.

Model	Meta-analysis C-index (95% CI)		Guangxi verification queue C-index (95% CI)	
	**3 years**	**5 years**	**3 years**	**5 years**
REACH-B	–	0.71 (0.70–0.73)	0.56 (0.37–0.75)	0.59 (0.47–0.70)
GAG-HCC	–	0.80 (0.78–0.82)	0.68 (0.53–0.83)	0.61 (0.50–0.73)
CUHCC	–	0.76 (0.75–0.78)	0.64 (0.46–0.83)	0.62 (0.50–0.73)

**FIGURE 6:**
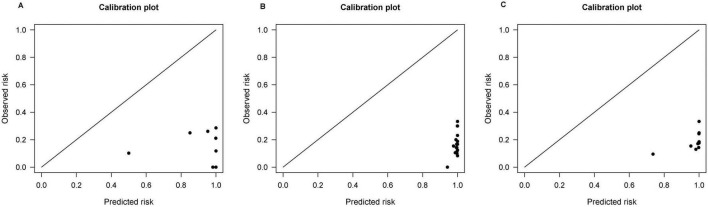
Calibration curves of REACH-B, GAGHCC, CUHCC, mPAGE-B, and PAGE-B models predicting time-independent hepatocellular carcinoma (HCC) risk.

External validation was performed on the REACH-B, GAGHCC, CUHCC, mPAGE-B, and PAGE-B models, and the time-independent discrimination was calculated, and the results are shown in Table 8. Except for the CUHCC model, the risk prediction ability of the remaining models for liver cancer in patients with chronic HBV infection in Guangxi was lower than the meta-analysis summary results, but significantly higher than the time-dependent discrimination, and the discrimination of each model was at a medium level; the calibration curve showed (Figure 6) that mPAGEB had the best accuracy.

**TABLE 8:** Time-dependent discrimination of REACH-B, GAGHCC, CUHC, mPAGE-B, and PAGE-B prediction models in the Guangxi chronic hepatitis B virus (HBV) infection cohort.

Model	Meta-analysis C-index (95% CI)		Guangxi verification queue C-index (95% CI)
	**3 years**	**5 years**	**Non-time dependent**
REACH-B	–	0.71 (0.70–0.73)	0.60 (0.54–0.65)
GAG-HCC	–	0.80 (0.78–0.82)	0.71 (0.64–0.77)
CUHCC	–	0.76 (0.75–0.78)	0.85 (0.81–0.89)
PAGE-B	–	0.79 (0.79–0.80)	0.70 (0.65–0.76)
mPAGE-B	–	0.77 (0.76–0.77)	0.75 (0.71–0.81)

## 4 Discussion

This study conducted a meta-analysis of six prediction models for the risk of HCC in patients with chronic HBV infection that had been externally validated more than five times. The results showed that the discrimination of the models included in the meta-analysis was at a moderate level, and the C-index values of the 5 years HCC risk ranged from 0.75 to 0.82. The mPAGE-B, GAG-HCC, and CAMD models had higher discrimination than other models. Guangxi is a high-incidence area for hepatitis B and related chronic liver diseases. Liver cancer is one of the cancers with a higher mortality rate in Guangxi. For a long time, the standardized mortality rate of liver cancer in Guangxi has ranked first in the country (24). In the patient cohort in Guangxi, the incidence of HCC increased year by year with the extension of follow-up time, with a cumulative incidence of 4% in 3 years and 11% in 5 years. The study also conducted independent external validation of the models included in the meta-analysis in the chronic HBV infection patient cohort in Guangxi and evaluated their predictive performance. It was found that these models had certain limitations in predicting the risk of HCC in chronic HBV infection patients in Guangxi, and their discrimination was significantly lower than the summary results of the meta-analysis. This shows that the prediction accuracy of the existing HBV-HCC risk prediction model in Guangxi may be affected by the local characteristics of the patients, the proportion of patients with cirrhosis, and the proportion of antiviral treatment. This study fills the research gap in the applicability of HCC prediction models in Guangxi and provides important reference value for HCC risk assessment in chronic HBV-infected patients in Guangxi. At the same time, it is of great significance to our team’s subsequent research on new HBV-HCC risk prediction models.

As age increases, the risk of HCC in HBV-infected patients also increases, and male patients are more likely to develop HCC than females (6). The six models included in the meta-analysis all use age and gender as predictors of HCC risk prediction. HBeAg and ALT are only used by the REACH-B model to predict the risk of HCC. Today, as the rate of antiviral treatment continues to increase, most patients become negative for HBeAg and remain within the normal range during antiviral treatment, which may weaken HBeAg and ALT. The role of ALT and even HBV DNA in HCC risk prediction has led to a significant decline in the discrimination of the REACH-B model in the external validation cohort. The REACH-B model based on modeling of patients without antiviral treatment may no longer be able to meet the current antiviral requirements. Viral therapy for HBV patients. In addition, the presence or absence of liver cirrhosis and/or PLT levels and/or ALB levels are included in all models as part of the HCC risk prediction model as indicators of liver cirrhosis, suggesting that liver cirrhosis is closely related to the occurrence of HCC, which is related to the risk of HCC. The natural history of the disease is consistent, but no model can incorporate the degree of liver cirrhosis quantification (such as LSM), which may reduce the discrimination of the model. The CAMD model also uses a history of diabetes as one of the predictors of HCC occurrence. This may be related to the fact that diabetes may promote the development of liver cirrhosis and changes in the microenvironment in the body. As a hepatologist, hepatologists should also pay attention to the causes of liver disease other than viral hepatitis. factors that aggravate sclerosis and comprehensively consider the treatment of various chronic diseases.

To date, there have been few systematic reviews or meta-analyses of established HCC risk prediction models and external validation of the corresponding models using external data. Most HCC risk prediction model modeling studies have established prediction models for various reasons. The discrimination and calibration performance are too ideal and cannot meet the actual clinical treatment needs of chronic hepatitis B patients in different regions (25). In our meta-analysis results, the discrimination of GAG-HCC and mPAGE-B models was better than that of other models, but there was no statistically significant difference in the discrimination between the two. Lee et al. (26) pointed out that the performance of mPAGE-B is similar to GAG-HCC and significantly higher than CU-HCC and REACH-B. Kim et al. (14) confirmed that the prediction performance of the mPAGE-B model is higher than other prediction models, but Chang et al. (45) believed in a study that the predictive performance of AASL score is better than mPAGE-B. These differences may be due to differences in race, treatment regimen, and disease status among participants in different external validation cohorts. Large heterogeneity. The three models REACH-B, GAGHCC, and CUHCC all showed good discrimination and calibration in the original modeling data set, but they were not reproduced in our study. This may be due to over-fitting during modeling. Related. In addition, our results show that the calibration curve for predicting 5 years HCC risk based on baseline results has a poor fit, while the calibration curve for immediate HCC risk prediction is better, indicating that the prediction model for predicting 5 years HCC risk based on baseline results is better. The accuracy is poor, which may be related to various uncertainties in the treatment process of patients with chronic HBV infection.

Our results also confirmed that the region of the patient population may be one of the sources of heterogeneity. In the regional subgroup analysis, except for the REACH-B model, the GAG-HCC, CUHCC, mPAGE-B, PAGE-B, and CAMD models had lower discrimination for HCC risk in Asians than in non-Asians (*P* < 0.05). The mPAGE-B model had the highest discrimination for predicting the 5 years HCC risk in Asians, but the prediction performance of each model was similar in non-Asians; this is consistent with the results of Wu et al. (27). In the bias risk subgroup analysis, the discrimination of the high-risk bias study subgroup was higher, which may be related to the overfitting of the model in the modeling cohort. Overfitting will make the discrimination and calibration performance of the established HCC risk prediction model too ideal. A meta-analysis study by Yang et al. (25) showed that only about 74% of HCC prediction model studies currently follow the reporting standards of multivariate prediction models for personal prognostic or diagnostic tools. In our study, almost all models did not explain how to handle missing data, and most external validation studies had less than 100 HCC outcome cases, which is the main reason for the high-risk bias. In addition, among the six models included in the analysis, only the REACH-B model provides a complete risk score and the corresponding risk probability of HCC occurrence, and can only simply divide patients into high, medium and low risk groups based on the risk score. It is unable to effectively convince patients to cooperate with the full-process management of chronic hepatitis B diagnosis and treatment, which may be the reason why the current HCC risk prediction model has not been recommended by the chronic hepatitis B diagnosis and treatment guidelines. The practicality of the currently constructed HCC risk prediction model in clinical work remains to be seen.

It is worth noting that our study has some limitations. First, because we could not obtain the original data included in the meta-analysis, we were unable to conduct a more detailed subgroup analysis to explore the predictive performance of the HCC risk prediction model in chronic HBV infection populations with different treatment status and disease status. Second, because most models were only validated by external cohorts for 5 years HCC risk prediction performance, there were few studies that validated the 1, 3, and 10 years HCC risk, which prevented us from analyzing and summarizing the models at different time points.

## 5 Conclusion

The published HBV-HCC risk prediction model has certain predictive value in predicting the risk of HCC in patients with chronic HBV infection, but it has certain geographical limitations and is restricted in its promotion and use. Its predictive ability in the population in Guangxi is obviously limited.

## Data Availability

The raw data supporting the conclusions of this article will be made available by the authors, without undue reservation.
