# Supplementary material for: Evaluating the predictive value of clinical models for HBV-related hepatocellular carcinoma: A meta-analysis
Source: Front Med (Lausanne). 2025 Feb 21;12:1529201. doi: 10.3389/fmed.2025.1529201 (PMC11885123; doi:10.3389/fmed.2025.1529201)
Supplement: Supplementary file 1 [file Table_1.docx]

Supplementary Material

# Supplementary Data

## Search strategy of PubMed database

1.Chronic Hepatitis B Virus Infection [Title/Abstract] OR Chronic Hepatitis B[Title/Abstract] OR Hepatitis B Virus Infection, Chronic [Title/Abstract] OR Hepatitis B, Chronic [Title/Abstract] OR "Hepatitis B, Chronic"[Mesh]

2.Validat*[Title/Abstract] OR develop*[Title/Abstract] OR Predict*[Title/Abstract] OR Rule*[Title/Abstract] OR *model*[Title/Abstract] OR Risk*[Title/Abstract] OR score*[Title/Abstract] OR Prognos*[Title/Abstract] OR Clinical*[Title/Abstract] OR characteristic*[Title/Abstract] OR nomogram*[Title/Abstract] OR Decision*[Title/Abstract] OR "Decision Support Systems, Clinical"[Mesh] OR "Clinical Decision Rules"[Mesh] OR "Forecasting"[Mesh] OR "Early Detection of Cancer"[Mesh] OR "Nomograms"[Mesh] OR "Models* "[Mesh]

3.ROC Curve[Title/Abstract] OR Stratification[Title/Abstract] OR Discrimina*[Title/Abstract] OR Calibration[Title/Abstract] OR c statistic[Title/Abstract] OR c index[Title/Abstract] OR c -statistic[Title/Abstract] OR c-index[Title/Abstract] OR Area under the curve[Title/Abstract] OR AUC[Title/Abstract] OR Multivariable[Title/Abstract] OR performance[Title/Abstract]

4.2 AND 3

5.Carcinoma*,Hepatocellular[Title/Abstract] OR Hepatocellular Carcinoma*[Title/Abstract] OR Liver Cell Carcinoma*[Title/Abstract] OR Liver Cancer*, Adult[Title/Abstract] OR Adult Liver Cancer*[Title/Abstract] OR Cancers*, Adult Liver[Title/Abstract] OR Carcinoma*, Liver Cell[Title/Abstract] OR Cell Carcinoma*, Liver[Title/Abstract] OR Hepatoma*[Title/Abstract] OR Carcinoma, Hepatocellular[Mesh]

6."Comment" [Publication Type] OR "Letter" [Publication Type] OR "Editorial" [Publication Type] OR "Review" [Publication Type] OR " Clinical Trial" [Publication Type]

7.(1 AND 4 AND 5) NOT 6

## Search strategy of Embase database

1.'chronic hepatitis b'/exp OR 'chronic hepatitis b virus infection':ti,ab,kw OR 'hepatitis b, chronic':ti,ab,kw

2.validat* OR develop*:ti,ab,kw OR predict*:ti,ab,kw OR rule*:ti,ab,kw OR model*:ti,ab,kw OR risk*:ti,ab,kw OR score*:ti,ab,kw OR prognos*:ti,ab,kw OR clinical*:ti,ab,kw OR characteristic*:ti,ab,kw OR nomogram*:ti,ab,kw OR decision*:ti,ab,kw OR 'clinical decision support system'/exp OR 'clinical decision rule'/exp OR forecasting* OR 'early cancer diagnosis'/exp OR 'nomogram'/exp OR 'statistical model'/exp

3.'roc curve':ti,ab,kw OR stratification:ti,ab,kw OR discrimina*:ti,ab,kw OR calibration:ti,ab,kw OR 'c statistic*':ti,ab,kw OR 'c index':ti,ab,kw OR 'c -statistic*':ti,ab,kw OR 'area under the curve':ti,ab,kw OR auc:ti,ab,kw OR multivariable:ti,ab,kw OR performance:ti,ab,kw

4.#2 AND #3

5.'liver cell carcinoma'/exp OR 'carcinoma in the liver':ti,ab,kw OR 'liver cell carcinoma':ti,ab,kw OR 'hepatocellular carcinoma*':ti,ab,kw

6.#1 AND #4 AND #5

7.#6 AND ('case control study'/de OR 'clinical article'/de OR 'comparative study'/de OR 'controlled study'/de OR 'diagnostic test accuracy study'/de OR 'major clinical study'/de OR 'observational study'/de OR 'predictive model'/de OR 'proportional hazards model'/de OR 'prospective study'/de OR 'randomized controlled trial'/de OR 'retrospective study'/de OR 'validation study'/de) AND 'article'/it

## Search strategy of Cochrane Database

1.MeSH descriptor: [Hepatitis B, Chronic] explode all trees

2.(Chronic Hepatitis B Virus Infection):ti,ab,kw

3.(Chronic Hepatitis B):ti,ab,kw

4.(Hepatitis B Virus Infection, Chronic):ti,ab,kw

5.1 or 2 or 3 or 4

6.MeSH descriptor: [Carcinoma, Hepatocellular] explode all trees

7.(Hepatocellular Carcinoma*):ti,ab,kw

8.(Hepatoma*):ti,ab,kw

9.(Adult Liver Cancers):ti,ab,kw

10.(Liver Cell Carcinoma*):ti,ab,kw

11.6 or 7 or 8 or 9 or 10

12.(Validat* or develop* or Predict* or Rule* or *model* or Risk* or score* or Prognos* or Clinical* or characteristic* or nomogram* or Decision*):ti,ab,kw

13.MeSH descriptor: [Decision Support Systems, Clinical] explode all trees

14.MeSH descriptor: [Clinical Decision Rules] explode all trees

15.MeSH descriptor: [Forecasting] explode all trees

16.MeSH descriptor: [Early Detection of Cancer] explode all trees

17.MeSH descriptor: [Nomograms] explode all trees

18.MeSH descriptor: [Models, Statistical] explode all trees

19.12 or 13 or 14 or 15 or 16 or 17 or 18

20.(ROC curve or Stratification or Discrimina* or Calibration or *index or *statistic or Area under the curve or AUC or Multivariable or performance):ti,ab,kw

21.19 and 20

22.5 and 11 and 21

## Search strategy of China Biomedical Literature Service System

1. "cancer, hepatocellular"[weight: expand] OR "hepatoma"[title: intelligent] OR "hepatocellular carcinoma"[title: intelligent] OR "liver cancer, adult"[title: intelligent]

2.("Hepatitis B"[Unweighted: Expanded]) OR ("Chronic Hepatitis B"[Abstract: Intelligent] OR "Chronic Hepatitis B"[Abstract: Intelligent] OR "HBV"[Abstract: Intelligent] OR "Liver Cirrhosis"[Abstract: Intelligent])

3. "Discrimination"[Abstract: Intelligent] OR "Calibration"[Abstract: Intelligent] OR "Sensitivity"[Abstract: Intelligent] OR "Specificity"[Abstract: Intelligent] OR "ROC Curve"[Abstract: Intelligent] OR "AUC"[Abstract: Intelligent] OR "C Statistic"[Abstract: Intelligent] OR "Index*"[Abstract: Intelligent]

4. "Prediction"[Title: Intelligence] OR "Score"[Title: Intelligence] OR "Risk"[Title: Intelligence] OR "Model"[Title: Intelligence]

5. (#4) AND (#3)

6. (#5) AND (#2) AND (#1)
